# Recent Development of LDL-Based Nanoparticles for Cancer Therapy

**DOI:** 10.3390/ph16010018

**Published:** 2022-12-23

**Authors:** Binghong He, Qiong Yang

**Affiliations:** Beijing Key Laboratory of Gene Resource and Molecular Development, College of Life Sciences, Beijing Normal University, Beijing 100875, China

**Keywords:** low-density lipoprotein, nanoparticle, cancer therapy

## Abstract

Low-density lipoprotein (LDL), a natural lipoprotein transporting cholesterol in the circulatory system, has been a possible drug carrier for targeted delivery. LDL can bind to the LDL receptor (LDLR) with its outside apolipoprotein B-100 and then enter the cell via LDLR-mediated endocytosis. This targeting function inspires researchers to modify LDL to deliver different therapeutic drugs. Drugs can be loaded in the surficial phospholipids, hydrophobic core, or apolipoprotein for the structure of LDL. In addition, LDL-like synthetic nanoparticles carrying therapeutic drugs are also under investigation for the scarcity of natural LDL. In addition to being a carrier, LDL can also be a targeting molecule, decorated to the surface of synthetic nanoparticles loaded with cytotoxic compounds. This review summarizes the properties of LDL and the different kinds of LDL-based delivery nanoparticles, their loading strategies, and the achievements of the recent anti-tumor advancement.

## 1. Introduction

In recent years, cancer has become a major public health problem worldwide, and the latest cancer statistics showed that cancer is the second leading cause of death in the United States [1]. Chemotherapy is one type of cancer treatment under investigation to improve mortality [2,3,4]. Although chemotherapy kills fast-growing cancer cells, the fast-growing and dividing healthy cells will also be affected [5]. Nanoparticles (NPs) carrying cytotoxic agents to cancer cells help lessen the toxicity of chemotherapy and provide a new strategy for targeted cancer therapy [6,7]. Traditional NPs are enriched in the tumor through the high permeability and retention effect (EPR effect) with their sizes, for instance, polymers, micelles, microspheres, liposomes, and so on [8,9,10]. Some of them have been approved by the FDA and clinical studies show that the utilization of these therapeutic particles significantly prevents the spread of liver cancer [9], gastric cancer [10] and breast cancer [11], and so on. However, most of these targeted NPs are passive NPs that would not actively target cancer cells. Active targeted NPs that can specifically target specific cancer cells with particular ligands or proteins might effectively increase the enrichment of NPs and reduce side effects [12].

Low-density lipoprotein (LDL) is one kind of nanoscale molecule that could be an actively targeted NP [13]. LDL is the cholesterol transporter in the body to deliver cholesterol to the cells expressing the LDL receptor (LDLR) [14]. LDLR is overexpressed on the hyperproliferative cells, especially cancer cells such as liver cancers, glioma cancers, and lung cancers [15,16,17,18]. LDLR would recognize the apolipoprotein B-100 (ApoB-100) on the LDL and then form the LDL-LDLR complex to start the endocytosis. ApoB-100 is the apolipoprotein on the LDL that confirms the targeting characteristic of this nanoscale molecule [19]. As an endogenous molecule, LDL would not trigger an immune response and reduce unnecessary stress for the body.

## 2. LDL and LDL Receptors

**Figure 1:**
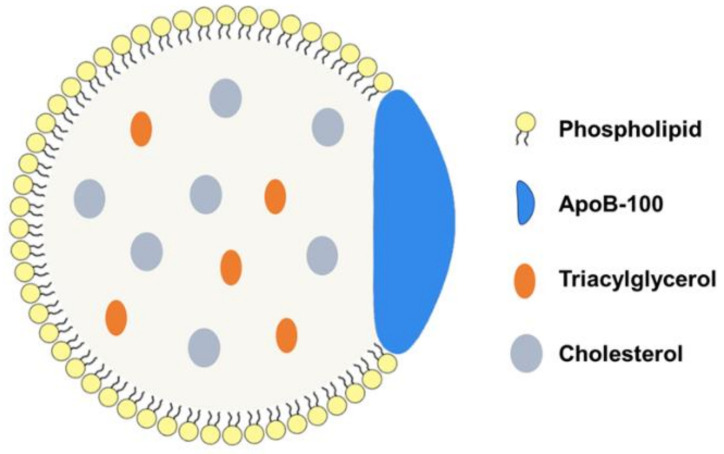
Schematic diagram of LDL structure. LDL is composed of phospholipid monolayers, cholesterols, triglycerides, and ApoB-100.

LDL is a spherical nanoscale molecule with a diameter of 18–25 nm [20], whose outer layer is a hydrophilic phospholipid monolayer, accompanied by ApoB-100 and free cholesterol, and the inner layer is a hydrophobic core composed of esterified cholesterol and triacylglycerol (Figure 1). The component of LDL is approximately 50% cholesterol, 25% protein, 20% phospholipids, and 5% triacylglycerol. ApoB-100 on the surface of LDL recognizes and binds to LDLR during endocytosis [21].

LDLR, a transmembrane glycoprotein consisting of 4536 amino acid residues, is one of the largest monomeric proteins [17]. LDLR is a widely expressed protein that absorbs approximately 70% of circulating LDLs via LDLR-mediated endocytosis [16]. During the endocytosis, the apoB-100 on the LDL would recognize the LDLR, and then LDL and LDLR form a complex. The complex enters the cell through the pits of the clathrin envelopes and is surrounded into a coated vesicle. Facilitated with the low pH environment of endosome and lysosome, LDL is digested to cholesterol and triglyceride while the LDLR returns to the cell membrane to restart another endocytosis [22].

**Figure 2:**
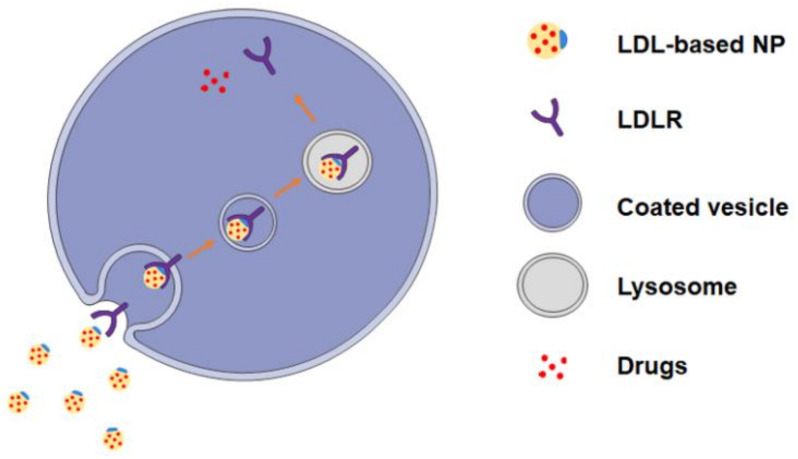
Schematic diagram of cellular uptake of LDL-based NPs loaded with drugs by LDLR-mediated endocytosis.

LDLs, as the crucial source of exogenous cholesterol, play a crucial role in providing cholesterol for cell proliferation. LDLR is highly expressed in malignant tumors such as gastric cancer [23], liver cancer [24], breast cancer [25], and leukemia [26], indicating that the intake of LDL might accelerate oncogenic processes. Caruso et al. found that, in rapidly proliferating tumor cells, the metabolic rate of LDLR significantly accelerated [27]. Given this function, Krieger et al. combined cytotoxic drugs with LDL to form LDL-based NPs and observed more accumulation of NPs in LDLR overexpressing tumor cells [28]. The intake of LDL-based NPs is dependent on the LDLR-mediated endocytosis, and the release of drugs is facilitated with lysosome (Figure 2). The targeting function of LDL-based NPs relies on the LDLR expression level but not the EPR effect; therefore, LDL-based NPs are suitable for malignancy including leukemia and solid tumor cells.

## 3. Categories of LDL-Based NPs

The affinity of ApoB-100 with LDLR results in the combination of LDL and LDLR, which facilitates the uptake of LDL. Thus, LDL particles containing apoB-100 can be developed as an active targeted carrier to transport therapeutic drugs. Another modification to utilize the LDL and LDLR interaction is to use the whole LDL as a wizard decorated on the surface of the NPs to target LDLR overexpressed cells. Therapeutic agents can be added to its comparatively large-load hydrophobic core, apolipoprotein, and monolayer phospholipid when LDL is used as carrier [29]. Because of the limited sources of native LDL, Nikanjam and collaborators used phosphatidylcholine, cholesterol oleic acid, ApoB-100 protein, or other synthetic peptides to develop synthesized LDL-like nanoparticles [16]. From then on, synthesized LDL-drugs nanoparticles are developed to deliver therapeutic drugs in vitro and in vivo. Here, we classify LDL-based drug-loaded particles into three types according to their source and function:

**Native LDL-drug nanoparticles (nLDL-drugs)** refer to nanoparticles composed of natural LDLs and cytotoxic drugs. **Synthesized LDL-drug nanoparticles (sLDL-drugs)** refer to nanoparticles that own the basic structure of LDL, coming from de novo synthesized LDL-like nanoparticles with therapeutic drugs loaded. **LDL decorated targeting nanoparticles (LDL-NPs)** refer to artificial nanoparticles formed by coupling native LDLs to the outer layer of the synthetic inorganic NPs carried therapeutic drugs.

We summarize the reconstruction methods of LDL-based nanoparticles and their recent achievements, which will provide the newest investigations of the LDL-based targeted delivery. LDL-based NPs owns the following advantages.

Firstly, LDL-based NPs are highly biologically safe. As a biological molecule, LDL has biocompatibility and good biodegradability. LDL will be degraded into recyclable units, including cholesterol, fatty acids, and amino acids in the lysosome.

Secondly, LDL-carriers can effectively avoid triggering the immune system, ensuring that the delivered drugs reach the target cells before the clearance.

Thirdly, LDL enhances the targeting function of anticancer drugs through LDLR-mediated endocytosis. The LDLR and LDL would form a complex and then enter cells through the clathrin internalization pathway. About 30–40% of LDL is cleared every day, and two-thirds of them are absorbed by receptor-mediated endocytosis.

Fourthly, LDL has a long circulation time (2–4 days), which can maintain the drug concentration in the body and prolong the residence of the drugs.

### 3.1. Native LDL-Drug Particles (nLDL-Drugs)

Commercial LDLs are mainly derived from native LDLs isolated from human or animal plasma. The native LDLs own the advantages of stable structure, immunogenicity-reduction, and superior targeting function. Usually, there are three strategies to form nLDL-drugs due to the structure of LDL, including phospholipid monolayer loading, protein loading, and core loading. The hydrophobic lipid core and amphiphilic phospholipids of LDLs can load with a mass of lipophilic and amphiphilic drugs, and the amino acid residues of ApoB-100 can be covalently bound to diagnostic ligands or therapeutic agents [30] (Table 1).

**Figure 3:**
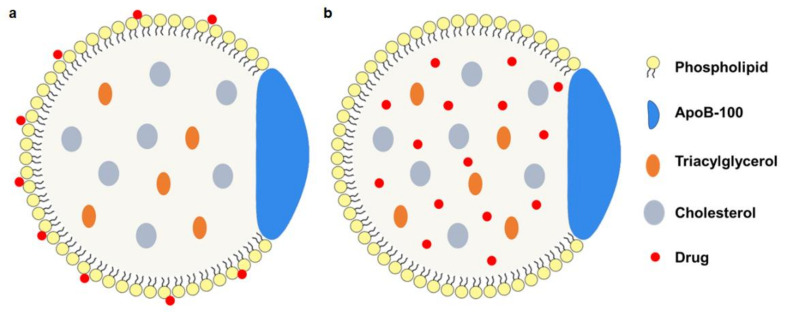
The modification strategy of nLDL-drugs particles. (**a**) drugs are inserted in the phospholipid monolayer; (**b**) drugs are loaded to the hydrophobic core.

**Phospholipid monolayer loading** This modification of the phosphate monolayer of LDL mainly relies on relatively weak interactions such as van der Waals forces or other non-covalent bonds. Usually, therapeutic drugs are inserted into the phospholipid monolayer [26] (Figure 3a). Because of the amphiphilic characteristic of the phospholipid, it is easier for therapeutic drugs with the amphiphilic structure to be inserted, while non-amphiphilic drugs require more steps to insert into the phospholipid. The nLDL-drugs from this way are relatively simple, uniform, and high-integrity.

**Apolipoprotein loading** This method involves the covalent binding of diagnostic or therapeutic drugs to ApoB-100. Lysine, arginine, tyrosine, and cysteine residues are used to couple amino acids on apolipoprotein residue [31], of which the lysine side chain is connected. To date, this method mainly delivers a contrast agent to monitor the biodistribution in the human body for its limited loading capacity. Meanwhile, the inactivation of ApoB-100 for extensive covalent modification [32] should be considered.

**Core loading** This method mainly refers to the reconstruction of therapeutic drugs on the core of LDL (Figure 3b). After the replacement of the non-polar core is achieved by freeze-drying or organic extraction [8], a drug/cholesterol mixture or drug-cholesterol conjugate [33] is used to form the reconstruction of the hydrophobic core. This method maintains the structure of phospholipids and apolipoprotein and ensures the binding activity of ApoB-100. Both hydrophilic and hydrophobic drugs could be delivered in this way. At the same time, sustained release and bioavailability are guaranteed.

### 3.2. Synthesized LDL-Drug Particles (sLDL-Drugs)

**Figure 4:**
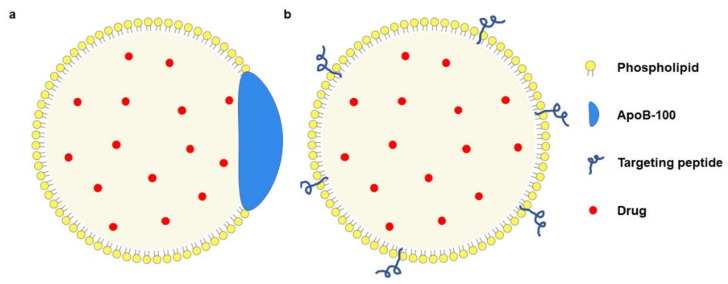
The modification strategy of sLDL-drugs. (**a**) targeting function is provided by ApoB-100; (**b**) targeting function is provided by biomimetic peptide.

The sLDL-drugs are mainly obtained by the solvent evaporation method [16] or the solvent emulsification method [34]. In addition, sLDL-drugs are usually composed of a purified lipid emulsion with LDLR recognition functions and therapeutic drugs. Antitumor drugs are encapsulated in a hydrophobic core (Figure 4a) [16,35] with mixed alcohol oleic acid. The outer layer is a single-layer shell composed of hydrophilic phospholipids, where the ApoB-100 is embedded. Sometimes, special proteins or polypeptides would replace the apoB-100 to bind to the LDLR binding domain (Figure 4b) [16]. Compared with nLDL-drugs, the sLDL-drugs obtained by this method are more structurally stable and overcome leakage during transportation.

### 3.3. LDL Decorated LDLR Targeting Nanoparticles NPs (LDL-NPs)

**Figure 5:**
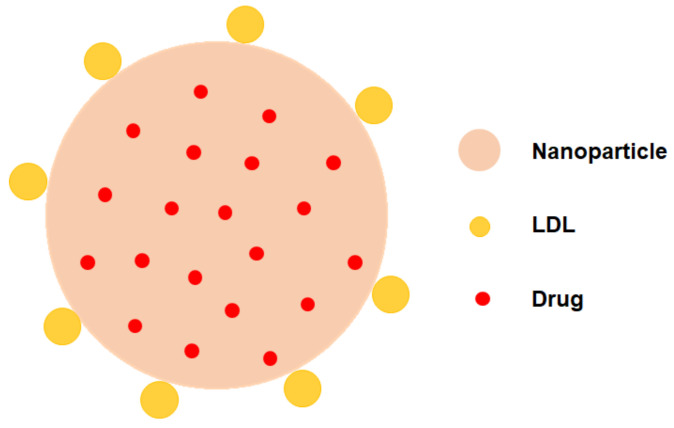
Model diagram of LDL-NPs. The NP core is loaded with small molecule drugs, and the LDL is attached to the outer layer of the NP as a targeting molecule.

Because of the good biocompatibility and biodegradability property of chitosan (CS) and silica nanoparticles (SLN), CS or SLN-based LDL-containing targeting nanoparticles have been constantly developed [36]. In LDL-modified NPs, LDL serves as the targeting molecule in therapy (Figure 5). Due to the large loading capacity of CS and SLN, multiple active pharmaceutical ingredients can be encapsulated in a single NP, thereby achieving synergistic therapy. LDL and synthetic nanoparticles are mainly combined with electrostatic interactions in this way.

## 4. Application of LDL-Based NPs in Cancer Therapy

### 4.1. nLDL-Drugs

Using natural LDL as a carrier to deliver therapeutic drugs has successfully inhibited the proliferation of melanoma, liver cancer, lung cancer, and so on [22,23]. There are several nLDL-drugs under investigation, and they made progress in cancer therapy (Table 2). Krieger et al. first designed r [25-HC-oleate] LDL to effectively reduce the activity of 3-hydroxy-3-methylglutaryl coenzyme A reductase in human fibroblasts, proving that constitutive LDL could selectively transport hydrophobic compounds to cells with LDLR [28]. Then, Masquelier et al. obtained an antitumor drug composed of a lipophilic derivation of doxorubicin (DNR) and LDL, termed DNR-LDL via lyophilization [26]. By investigating the in vivo fate of the complex, it was found that the DNR-LDL was quite similar to native LDL, and the high LDLR activity of the cancer cells resulted in the accumulation of DNR-LDL. Samadi-Baboli et al. confirmed that LDL-based nanoparticles were dependent on LDLR-mediated endocytosis, and the potency of lipophilic cytotoxic drugs against tumors was improved when combined with LDL [37]. Lo et al. prepared LDL-Doxorubicin (LDL-DOX) and injected it to liver cancer-bearing mice. It is found that LDL-DOX could selectively accumulate in the cancer cells, indicating that cancer cells had elevated expression of LDLR and more intake of LDL [38].

In addition, to widely incorporate cytotoxic drugs into LDL, LDL could also carry low-toxicity docosahexaenoic acid (DHA), an omega-3 polyunsaturated fatty acid, to improve cardiovascular health and prevent cancer. Reynolds et al. constructed LDL-DHA nanoparticles and found that the effective therapeutic dose of LDL-DHA on cancer cells does not influence normal cells, which may rely on the active lipid peroxidation and selective induction of reactive oxygen for cancer cells [8]. Yang et al. further evaluated the LDL-DHA on human liver cancer stem cells (CSC) and found that CSCs had a lower survival rate than normal cancer cells for the different LDLR expression [39], verifying LDL as a suitable delivery platform for drug-resistant cancer stem cells. Wen et al. achieved significant disease burden reduction by using synthetic LDL-DHA for liver cancer in situ [17]. Malik et al. combined LDL-DHA with pulsed-focused ultrasound technology to deliver LDL-DHA locally to the brain, and highly concentrated LDL-DHA in the treatment area was observed [40].

**Table 2:** nLDL-drugs and applications.

Category	Drugs	Indications	Reference
DNR-LDL	Doxorubicin	Leukemia cells	[26]
m-LDL	Cytotoxic compound 25	Lung fibroblasts	[41]
OL-NME-LDL	Elliptinium-oleate	B16 melanoma	[37]
Paclitaxel-LDL	Paclitaxel	Leukemia cells	[26]
LDL-DOX	Doxorubicin	HepG2 cells	[42]
LDL-DOX	Doxorubicin	R-HepG2 cells	[38]
r-Pc-LDL-FA	Tetra-t-butyl-silicon phthalocyanine	KB cells, HT-1080 cells, and HepG2 cells	[30]
Hyp-LDL	Hypericin	U87-MG cells	[43]
LDL-DHA	Docosahexaenoic acid	Hepatoma cells (H4IIE)	[15]
LDL-DNA	Docosahexaenoic acid	Fibroblasts	[44]
CaP@LDL	STAT3-decoyodns	HepG2 and PLC/PRF/5 cells	[45]
DOX-LDL	Doxorubicin	A549 cells	[18]
LDL-DHA	Docosahexaenoic acid	HuH-7 and HepG2 cells	[39]
LDL-DHA	Docosahexaenoic acid	TIB-75 cells	[8]

### 4.2. sLDL-Drugs

Since LDL source is scarce, synthetic LDL-like particles gradually become prevalent in reconstructed LDL-based NPs. The research on the combination of synthetic LDL (sLDL) and their different therapeutic preparations develop rapidly and achieve superior therapeutic effects (Table 3).

Baillie et al. prepared sLDL by combining lipid microemulsion with amphiphilic peptides containing apolipoprotein B receptor domain. This sLDL was taken up via LDLR-mediated endocytosis and could support the proliferation of U937 in culture [46]. Nikanjam et al. synthesized a new nanoparticle composed of an LDL-like shell with Paclitaxel oleate (PO) loaded, which was termed nLDL-PO, and demonstrated that only 6 h was needed for nLDL-PO to deliver Paclitaxel (PTX) to glioma cells via LDLR-mediated endocytosis [17]. Kim et al. developed biocompatible anti-cancer paclitaxel therapeutic solid lipid nanoparticles (PtSLNs) by containing paclitaxel in the core and modifying PEG on the surface to connect the tumor-targeting ligand [47]. As excepted, PtSLNs demonstrated a better targeting effect than the clinically free Taxol. Su et al. prepared sLDL to encapsulate paclitaxel-alpha linolenic acid (PALA) for tumor therapy. PTX-loaded nano-drug PALA-sLDL had a suitable size (approximately 66 nm) and high loading efficiency, and a good tumor growth inhibitory effect in U87 MG mice [48]. Qian et al. connected the lipid binding motif of apoB-100 to one end of PEG and introduced folate acid (FA) as a tumor-targeting moiety to the other end of PEG, constructing targeted folate receptor (FR) LDL bionic nanoparticles [34]. The uptake of these nanoparticles in FR-overexpressing tumor cells (HeLa cells) was much higher than that of FR-deficient tumor cells (A549 cells), at the same time producing a very significant anti-tumor efficiency in M109 tumor-bearing mice. Similar folic acid functionalized LDL biomimetic particles achieved co-delivery of anticancer drugs and superparamagnetic nanocrystals in MCF cells [25].

**Table 3:** sLDL-drugs and applications.

Category	Drugs	Indications	Reference
nLDL-PO	Paclitaxel oleate	GBM cells	[17]
siRNA-PEG/SLN	siRNA	PC3 cells	[49]
Targeted PtSLNs	Paclitaxel	NCI-H1975, NCI-H1650, NCI-H520, PC9	[47]
FA-mpLNPs	Iron oxide nanocrystals	MCF-7	[25]
PALA-sLDL	Paclitaxel-alpha linolenic acid	U87 MG, HepG2	[48]
FPLM NPs	Paclitaxel	HeLa, A549	[34]
AODN	Pro-doxorubicin	4T1	[35]
Lf-mNLC	Curcumin	BCECs	[50]

### 4.3. LDL-NPs

LDL-modified NPs are prepared using chitosan (CS) or solid lipid nanoparticles (SLN) as the carriers and LDL molecules as targeting ligands. This nanoparticle has a better targeting capacity to LDLR overexpressed cells and higher loading ability (Table 4). Currently, LDL-NPs are applied in the treatment of liver and breast cancer. Zhang et al. first synthesized N-succinyl-chitosan (NSC) as the center part of NPs and then loaded osthole to obtain Ost/LDL-NSC-NPs. Excellent proliferation inhibition could be observed when applying these NPs to HepG2 cells [36]. Based on this, Zhu et al. used LDL-SCS-NPs to deliver siRNA and doxorubicin to liver cancers, providing a novel and effective way for the co-delivery of genes and chemotherapeutic drugs [51]. Ao et al. developed the SLN-based docetaxel (DTX) and thalidomide (TDD) co-delivery system and found that these NPs had strong targeting properties [52]. Ye et al. used SLN-based LDL-containing NPs to co-deliver sorafenib (Sor) and doxorubicin (Dox). Obvious tumor inhibition was observed in vitro and in vivo [53]. Wang et al. developed co-drug lipid nanoparticles LD-SDN to transport sorafenib and dihydroartemisinin to liver cancer cells, revealing excellent apoptosis induction [24].

Yang et al. developed a binary copolymer system based on N-succinyl chitosan lipoic acid micelles to co-deliver the siRNA and paclitaxel [54] to inhibit the growth of breast cancer cells in vitro and in vivo. Zhu et al. prepared PTX and siRNA co-loading drug siRNA-PTX/LDL-NSC-SS-UA and made significant progress in overcoming multidrug resistance [55]. Pan et al. used the SLN-designed and synthesized indocyanine green multifunctional platform (LDL/SLN) to target breast cancer cells, which provided new ideas for the development of photothermal therapy for breast cancer [56].

**Table 4:** LDL-NPs and applications.

Category	Drugs	Indications	Reference
Ost/LDL-NSC-NPs	Osthole	HepG2 cells	[36]
Dox-siRNA/LDL-SCS-NPs	Dox siRNA	HepG2, H22	[51]
PTX-siRNA/LDL-NSC-LA micelles	MDR1 siRNA and paclitaxel	MCF-7 cells	[54]
LDL/SLN/DTX/TDD	Docetaxel (DTX), Thalidomide (TDD)	HepG2 cells	[52]
LDL/SLN/Adr	Adriamycin	Colorectal cancer	[57]
LDL-SLN/Sor/Dox	Sorafenib, Doxorubicin	HepG2 cells	[53]
LD-SDN	Sorafenib, Dihydroartemisinin	HepG2 cells	[24]
LDL/SLN/ICG	Sorafenib, Dihydroartemisinin	MCF-7 cells	[56]

Nowadays, more and more studies are focused on bionic nanoparticles. Compared with the modification of nLDL-drugs particles, LDL-NPs can further save costs, improve plasticity, and provide more modification methods and materials. These biomimetic materials have larger particle sizes and more target candidates to interact with cancer cells. Although the nLDL-drugs have been investigated for a long time, and the experimental protocols are experienced, there are still some challenges to uniformity and integrity. Therefore, more and more investigation of sLDL-drugs and LDL-NPs is prevalent. Here, we compare the advantages and disadvantages of these three particles (Table 5).

## 5. Conclusions and Future Prospects

LDL has the characteristics of small size, amphiphilic molecular surface, receptor-mediated internalization, and long circulation time. With these functions, therapeutic drugs can be combined with LDL to target LDLR-overexpressed tumor cells. As an ideal carrier and effective ligand for targeted therapy, LDL-based drug-loaded nanoparticles further promote the development of targeted delivery and expand the application of LDL in cancer therapy. Most targeting NPs are designed for solid tumors but not hematological malignancies. This is because hematological oncology is different from solid ones in that leukemia cells might be all around the body, while solid tumor cells usually locate in a particular tissue. LDL-based NPs depend on LDLR-endocytosis to effectively target cancer cells and thus can be utilized in leukemia and solid tumors. By comparing the degradation efficiency of monocyte 125I-LDL isolated from healthy individuals and leukemia patients, Viitols et al. found that primary leukemia cells had a higher degradation rate of 125I-LDL [59]. Then, Zhou et al. found that the intake of sLDL was inversely proportional to the degree of cell differentiation by using synthetic LDL particles to target leukemia cell lines and CML patient stem/progenitor cells [60]. Hence, LDL could be a potential drug delivery carrier for leukemia disease.

Although the research on LDL-based NPs has made many achievements, there are still limitations to clinical advancement. The limits are the sources of LDL, the complex processing requirements, and the trigger of atherosclerosis in the body. sLDL-drugs and LDL-NPs are derived from non-plasma-separated LDL, overcoming the problem of resource limitations. In addition, they still restore the targeting characteristics of LDL and keep the simple and controllable synthesis process. Due to the larger particle diameter, the loading capacity also increases. Meanwhile, LDL-based targeting NPs not only target solid tumors but also hematological malignancy. More importantly, these NPs have stronger targeting function to cancer stem cells or drug-resistant cells for these cells overexpressing LDLR. In short, LDL-based nanoparticles have good application prospects in cancer-targeted therapy.

## Figures and Tables

**Table 1:** Advantages and disadvantages of different modification strategies.

Strategy	Advantage	Disadvantages
Phospholipid monolayer loading	Simple operation Larger drug load	Drug Amphiphile Easy leakage
Apolipoprotein loading	Simple operation	ApoB-100 inactivation Less drug load
Core loading	Large drug load Less damage to the shell	Cumbersome operation Drug hydrophobicity

**Table 5:** Comparison of LDL-based NPs.

Strategy	Advantages	Disadvantages
nLDL-drugs	Biocompatibility, biodegradability Targeting Long half-life	Scarce raw materials and high cost [18] Poor stability, harsh storage conditions [8]
sLDL-drugs	Low cost High loading capacity Targeting peptides to further improve targeting	Complex process requirements [17] Not very targeting capacity [58]
LDL-NPs	Low cost Simple protocol Diversity targeting moiety	Large size [24] Exogenous material [56]

## Data Availability

Data sharing not applicable.

## References

[1] Siegel R.L., Miller K.D., Fuchs H.E., Jemal A. (2022). Cancer Statistics, 2022. CA. Cancer J. Clin..

[2] Sparano J.A., Gray R.J., Makower D.F., Albain K.S., Saphner T.J., Badve S.S., Wagner L.I., Kaklamani V.G., Keane M.M., Gomez H.L. (2020). Clinical Outcomes in Early Breast Cancer With a High 21-Gene Recurrence Score of 26 to 100 Assigned to Adjuvant Chemotherapy Plus Endocrine Therapy: A Secondary Analysis of the TAILORx Randomized Clinical Trial. JAMA Oncol..

[3] Moy B., Rumble R.B., Come S.E., Davidson N.E., Di Leo A., Gralow J.R., Hortobagyi G.N., Yee D., Smith I.E., Chavez-MacGregor M. (2021). Chemotherapy and Targeted Therapy for Patients With Human Epidermal Growth Factor Receptor 2-Negative Metastatic Breast Cancer That Is Either Endocrine-Pretreated or Hormone Receptor-Negative: ASCO Guideline Update. J. Clin. Oncol..

[4] Lu S., Wu L., Jian H., Chen Y., Wang Q., Fang J., Wang Z., Hu Y., Sun M., Han L. (2022). Sintilimab plus Bevacizumab Biosimilar IBI305 and Chemotherapy for Patients with EGFR-Mutated Non-Squamous Non-Small-Cell Lung Cancer Who Progressed on EGFR Tyrosine-Kinase Inhibitor Therapy (ORIENT-31): First Interim Results from a Randomised, Double-Blind, Multicentre, Phase 3 Trial. Lancet. Oncol..

[5] Schwartzberg L.S., Modiano M.R., Rapoport B.L., Chasen M.R., Gridelli C., Urban L., Poma A., Arora S., Navari R.M., Schnadig I.D. (2015). Safety and Efficacy of Rolapitant for Prevention of Chemotherapy-Induced Nausea and Vomiting after Administration of Moderately Emetogenic Chemotherapy or Anthracycline and Cyclophosphamide Regimens in Patients with Cancer: A Randomised, Active-Controlled, Double-Blind, Phase 3 Trial. Lancet. Oncol..

[6] An X., Zhu A., Luo H., Ke H., Chen H., Zhao Y. (2016). Rational Design of Multi-Stimuli-Responsive Nanoparticles for Precise Cancer Therapy. ACS Nano.

[7] Aikins M.E., Xu C., Moon J.J. (2020). Engineered Nanoparticles for Cancer Vaccination and Immunotherapy. Acc. Chem. Res..

[8] Reynolds L., Mulik R.S., Wen X., Dilip A., Corbin I.R. (2014). Low-Density Lipoprotein-Mediated Delivery of Docosahexaenoic Acid Selectively Kills Murine Liver Cancer Cells. Nanomedicine.

[9] Tabernero J., Shapiro G.I., LoRusso P.M., Cervantes A., Schwartz G.K., Weiss G.J., Paz-Ares L., Cho D.C., Infante J.R., Alsina M. (2013). First-in-Humans Trial of an RNA Interference Therapeutic Targeting VEGF and KSP in Cancer Patients with Liver Involvement. Cancer Discov..

[10] Xu X., Wang L., Xu H.Q., Huang X.E., Qian Y.D., Xiang J. (2013). Clinical Comparison between Paclitaxel Liposome (Lipusu®) and Paclitaxel for Treatment of Patients with Metastatic Gastric Cancer. Asian Pac. J. Cancer Prev..

[11] Awada A., Garcia A.A., Chan S., Jerusalem G.H.M., Coleman R.E., Huizing M.T., Mehdi A., O’Reilly S.M., Hamm J.T., Barrett-Lee P.J. (2013). Two Schedules of Etirinotecan Pegol (NKTR-102) in Patients with Previously Treated Metastatic Breast Cancer: A Randomised Phase 2 Study. Lancet. Oncol..

[12] Wicki A., Witzigmann D., Balasubramanian V., Huwyler J. (2015). Nanomedicine in Cancer Therapy: Challenges, Opportunities, and Clinical Applications. J. Control. Release.

[13] McConathy W.J., Paranjape S., Mooberry L., Buttreddy S., Nair M., Lacko A.G. (2011). Validation of the Reconstituted High-Density Lipoprotein (RHDL) Drug Delivery Platform Using Dilauryl Fluorescein (DLF). Drug Deliv. Transl. Res..

[14] Taskinen M.R. (2003). LDL-Cholesterol, HDL-Cholesterol or Triglycerides - Which Is the Culprit?. Diabetes Res. Clin. Pract..

[15] Wen X., Reynolds L., Mulik R.S., Kim S.Y., Van Treuren T., Nguyen L.H., Zhu H., Corbin I.R. (2016). Hepatic Arterial Infusion of Low-Density Lipoprotein Docosahexaenoic Acid Nanoparticles Selectively Disrupts Redox Balance in Hepatoma Cells and Reduces Growth of Orthotopic Liver Tumors in Rats. Gastroenterology.

[16] Nikanjam M., Blakely E.A., Bjornstad K.A., Shu X., Budinger T.F., Forte T.M. (2007). Synthetic Nano-Low Density Lipoprotein as Targeted Drug Delivery Vehicle for Glioblastoma Multiforme. Int. J. Pharm..

[17] Nikanjam M., Gibbs A.R., Hunt C.A., Budinger T.F., Forte T.M. (2007). Synthetic Nano-LDL with Paclitaxel Oleate as a Targeted Drug Delivery Vehicle for Glioblastoma Multiforme. J. Control. Release.

[18] Zhu C., Pradhan P., Huo D., Xue J., Shen S., Roy K., Xia Y. (2017). Reconstitution of Low-Density Lipoproteins with Fatty Acids for the Targeted Delivery of Drugs into Cancer Cells. Angew. Chem. Int. Ed. Engl..

[19] Young S.G. (1990). Recent Progress in Understanding Apolipoprotein B. Circulation.

[20] Zhang Y., Sun T., Jiang C. (2018). Biomacromolecules as Carriers in Drug Delivery and Tissue Engineering. Acta Pharm. Sin. B.

[21] Damiano M.G., Mutharasan R.K., Tripathy S., McMahon K.M., Thaxton C.S. (2013). Templated High Density Lipoprotein Nanoparticles as Potential Therapies and for Molecular Delivery. Adv. Drug Deliv. Rev..

[22] Jeon H., Blacklow S.C. (2005). Structure and Physiologic Function of the Low-Density Lipoprotein Receptor. Annu. Rev. Biochem..

[23] Li C., Zhang J., Wu H., Li L., Yang C., Song S., Peng P., Shao M., Zhang M., Zhao J. (2017). Lectin-like Oxidized Low-Density Lipoprotein Receptor-1 Facilitates Metastasis of Gastric Cancer through Driving Epithelial-Mesenchymal Transition and PI3K/Akt/GSK3β Activation. Sci. Rep..

[24] Wang Z., Duan X., Lv Y., Zhao Y. (2019). Low Density Lipoprotein Receptor (LDLR)-Targeted Lipid Nanoparticles for the Delivery of Sorafenib and Dihydroartemisinin in Liver Cancers. Life Sci..

[25] Lee J.Y., Kim J.H., Bae K.H., Oh M.H., Kim Y., Kim J.S., Park T.G., Park K., Lee J.H., Nam Y.S. (2015). Low-Density Lipoprotein-Mimicking Nanoparticles for Tumor-Targeted Theranostic Applications. Small.

[26] Masquelier M., Vitols S., Palsson M., Mars U., Larsson B.S., Peterson C.O. (2000). Low density lipoprotein as a carrier of cytostatics in cancer chemotherapy: Study of stability of drug-carrier complexes in blood. J. Drug Target..

[27] Caruso M.G., Notarnicola M., Cavallini A., Guerra V., Misciagna G., Di Leo A. (1993). Demonstration of Low Density Lipoprotein Receptor in Human Colonic Carcinoma and Surrounding Mucosa by Immunoenzymatic Assay. Ital. J. Gastroenterol..

[28] Krieger M., Goldstein J.L., Brown M.S. (1978). Receptor-Mediated Uptake of Low Density Lipoprotein Reconstituted with 25-Hydroxycholesteryl Oleate Suppresses 3-Hydroxy-3-Methylglutaryl-Coenzyme A Reductase and Inhibits Growth of Human Fibroblasts. Proc. Natl. Acad. Sci. USA.

[29] Chu H.L., Cheng T.M., Chen H.W., Chou F.H., Chang Y.C., Lin H.Y., Liu S.Y., Liang Y.C., Hsu M.H., Wu D.S. (2013). Synthesis of Apolipoprotein B Lipoparticles to Deliver Hydrophobic/Amphiphilic Materials. ACS Appl. Mater. Interfaces.

[30] Zheng G., Chen J., Li H., Glickson J.D. (2005). Rerouting Lipoprotein Nanoparticles to Selected Alternate Receptors for the Targeted Delivery of Cancer Diagnostic and Therapeutic Agents. Proc. Natl. Acad. Sci. USA.

[31] Bergt C., Fu X., Huq N.P., Kao J., Heinecke J.W. (2004). Lysine Residues Direct the Chlorination of Tyrosines in YXXK Motifs of Apolipoprotein A-I When Hypochlorous Acid Oxidizes High Density Lipoprotein. J. Biol. Chem..

[32] Versluis A.J., Van Geel P.J., Oppelaar H., Van Berkel T.J.C., Bijsterbosch M.K. (1996). Receptor-Mediated Uptake of Low-Density Lipoprotein by B16 Melanoma Cells in Vitro and in Vivo in Mice. Br. J. Cancer.

[33] Jin H., Lovell J.F., Chen J., Ng K., Cao W., Ding L., Zhang Z., Zheng G. (2011). Cytosolic Delivery of LDL Nanoparticle Cargo Using Photochemical Internalization. Photochem. Photobiol. Sci..

[34] Qian J., Xu N., Zhou X., Shi K., Du Q., Yin X., Zhao Z. (2019). Low Density Lipoprotein Mimic Nanoparticles Composed of Amphipathic Hybrid Peptides and Lipids for Tumor-Targeted Delivery of Paclitaxel. Int. J. Nanomedicine.

[35] Li W., Fu J., Ding Y., Liu D., Jia N., Chen D., Hu H. (2019). Low Density Lipoprotein-Inspired Nanostructured Lipid Nanoparticles Containing pro-Doxorubicin to Enhance Tumor-Targeted Therapeutic Efficiency. Acta Biomater..

[36] Zhang C.G., Zhu Q.L., Zhou Y., Liu Y., Chen W.L., Yuan Z.Q., Yang S.D., Zhou X.F., Zhu A.J., Zhang X.N. (2014). N-Succinyl-Chitosan Nanoparticles Coupled with Low-Density Lipoprotein for Targeted Osthole-Loaded Delivery to Low-Density Lipoprotein Receptor-Rich Tumors. Int. J. Nanomedicine.

[37] Samadi-Baboli M., Favre G., Canal P., Soula G. (1993). Low Density Lipoprotein for Cytotoxic Drug Targeting: Improved Activity of Elliptinium Derivative against B16 Melanoma in Mice. Br. J. Cancer.

[38] Lo E.H.K., Ooi V.E.L., Fung K.P. (2002). Circumvention of Multidrug Resistance and Reduction of Cardiotoxicity of Doxorubicin in Vivo by Coupling It with Low Density Lipoprotein. Life Sci..

[39] Yang J., Gong Y., Sontag D.P., Corbin I., Minuk G.Y. (2018). Effects of Low-Density Lipoprotein Docosahexaenoic Acid Nanoparticles on Cancer Stem Cells Isolated from Human Hepatoma Cell Lines. Mol. Biol. Rep..

[40] Mulik R.S., Bing C., Ladouceur-Wodzak M., Munaweera I., Chopra R., Corbin I.R. (2016). Localized Delivery of Low-Density Lipoprotein Docosahexaenoic Acid Nanoparticles to the Rat Brain Using Focused Ultrasound. Biomaterials.

[41] Lundberg B. (1987). Preparation of Drug-Low Density Lipoprotein Complexes for Delivery of Antitumoral Drugs via the Low Density Lipoprotein Pathway. Cancer Res..

[42] Chu A.C.Y., Tsang S.Y., Lo E.H.K., Fung K.P. (2001). Low Density Lipoprotein as a Targeted Carrier for Doxorubicin in Nude Mice Bearing Human Hepatoma HepG2 Cells. Life Sci..

[43] Huntosova V., Buzova D., Petrovajova D., Kasak P., Nadova Z., Jancura D., Sureau F., Miskovsky P. (2012). Development of a New LDL-Based Transport System for Hydrophobic/Amphiphilic Drug Delivery to Cancer Cells. Int. J. Pharm..

[44] Khan Z., Hawtrey A.O., Ariatti M. (2003). New Cationized LDL-DNA Complexes: Their Targeted Delivery to Fibroblasts in Culture. Drug Deliv. J. Deliv. Target. Ther. Agents.

[45] Shi K., Xue J., Fang Y., Bi H., Gao S., Yang D., Lu A., Li Y., Chen Y., Ke L. (2017). Inorganic Kernel-Reconstituted Lipoprotein Biomimetic Nanovehicles Enable Efficient Targeting “Trojan Horse” Delivery of STAT3-Decoy Oligonucleotide for Overcoming TRAIL Resistance. Theranostics.

[46] Baillie G., Owens M.D., Halbert G.W. (2002). A Synthetic Low Density Lipoprotein Particle Capable of Supporting U937 Proliferation in Vitro. J. Lipid Res..

[47] Kim J.H., Kim Y., Bae K.H., Park T.G., Lee J.H., Park K. (2015). Tumor-Targeted Delivery of Paclitaxel Using Low Density Lipoprotein-Mimetic Solid Lipid Nanoparticles. Mol. Pharm..

[48] Su H.T., Li X., Liang D.S., Qi X.R. (2016). Synthetic Low-Density Lipoprotein (SLDL) Selectively Delivers Paclitaxel to Tumor with Low Systemic Toxicity. Oncotarget.

[49] Kim H.R., Kim I.K., Bae K.H., Lee S.H., Lee Y., Park T.G. (2008). Cationic Solid Lipid Nanoparticles Reconstituted from Low Density Lipoprotein Components for Delivery of SiRNA. Mol. Pharm..

[50] Meng F., Asghar S., Gao S., Su Z., Song J., Huo M., Meng W., Ping Q., Xiao Y. (2015). A Novel LDL-Mimic Nanocarrier for the Targeted Delivery of Curcumin into the Brain to Treat Alzheimer’s Disease. Colloids Surf. B Biointerfaces.

[51] Zhu Q.L., Zhou Y., Guan M., Zhou X.F., Yang S.D., Liu Y., Chen W.L., Zhang C.G., Yuan Z.Q., Liu C. (2014). Low-Density Lipoprotein-Coupled N-Succinyl Chitosan Nanoparticles Co-Delivering SiRNA and Doxorubicin for Hepatocyte-Targeted Therapy. Biomaterials.

[52] Ao M., Xiao X., Ao Y. (2018). Low density lipoprotein modified silica nanoparticles loaded with docetaxel and thalidomide for effective chemotherapy of liver cancer. Braz. J. Med. Biol. Res. Rev. Bras. Pesqui. Med. E Biol..

[53] Ye J., Zhang R., Chai W., Du X. (2018). Low-Density Lipoprotein Decorated Silica Nanoparticles Co-Delivering Sorafenib and Doxorubicin for Effective Treatment of Hepatocellular Carcinoma. Drug Deliv..

[54] Yang S.D., Zhu W.J., Zhu Q.L., Chen W.L., Ren Z.X., Li F., Yuan Z.Q., Li J.Z., Liu Y., Zhou X.F. (2017). Binary-Copolymer System Base on Low-Density Lipoprotein-Coupled N-Succinyl Chitosan Lipoic Acid Micelles for Co-Delivery MDR1 SiRNA and Paclitaxel, Enhances Antitumor Effects via Reducing Drug. J. Biomed. Mater. Res. B Appl. Biomater..

[55] Zhu W.J., Yang S.D., Qu C.X., Zhu Q.L., Chen W.L., Li F., Yuan Z.Q., Liu Y., You B.G., Zhang X.N. (2017). Low-Density Lipoprotein-Coupled Micelles with Reduction and PH Dual Sensitivity for Intelligent Co-Delivery of Paclitaxel and SiRNA to Breast Tumor. Int. J. Nanomedicine.

[56] Pan H., Sun Y., Cao D., Wang L. (2020). Low-Density Lipoprotein Decorated and Indocyanine Green Loaded Silica Nanoparticles for Tumor-Targeted Photothermal Therapy of Breast Cancer. Pharm. Dev. Technol..

[57] Shi G., Li J., Yan X., Jin K., Li W., Liu X., Zhao J., Shang W., Zhang R. (2019). Low-Density Lipoprotein-Decorated and Adriamycin-Loaded Silica Nanoparticles for Tumor-Targeted Chemotherapy of Colorectal Cancer. Adv. Clin. Exp. Med..

[58] Liu M., Li W., Larregieu C.A., Cheng M., Yan B., Chu T., Li H., Mao S.J. (2014). Development of Synthetic Peptide-Modified Liposomes with LDL Receptor Targeting Capacity and Improved Anticancer Activity. Mol. Pharm..

[59] Vitols S., Gahrton G., Ost A., Peterson C. (1984). Elevated Low Density Lipoprotein Receptor Activity in Leukemic Cells With Monocytic Differentiation. Blood.

[60] Zhou P., Hatziieremia S., Elliott M.A., Scobie L., Crossan C., Michie A.M., Holyoake T.L., Halbert G.W., Jørgensen H.G. (2010). Uptake of Synthetic Low Density Lipoprotein by Leukemic Stem Cells—A Potential Stem Cell Targeted Drug Delivery Strategy. J. Control. Release.

